# Notes and News

**Published:** 1906-02-24

**Authors:** 


### Notes and News.

Surgeon-General Spencer, late of the Indian forces, has been appointed Honorary Surgeon to the King.

The 76th meeting of the British Association will open at York on August 1. The President-elect is Professor Ray Lankester.

St. Mary's Hospital has received an anonymous donation of £1,000 towards the sum required to enable the Clarence Memorial wing to be furnished and opened for patients.

It is stated that of 79 theses for the degree of Doctor of Medicine presented to the Universities of Geneva and Lausanne last year, 42 were offered by women.

The total cost of the typhoid fever epidemic at Lincoln last year was £12,407. The number of patients treated in the hospitals was 424, the aggregate number of days under treatment being 19,682.

The Committee of Cardiff Infirmary have decided to remodel and enlarge the out-patients' department of that institution, the present accommodation being wholly unfitted and insufficient for the purpose.

The 54th anniversary of the foundation of the Hospital for Sick Children in Great Ormond Street was marked last week by the opening of a new ward to accommodate between 20 and 30 patients, and a suite of isolation wards at the top of the south wing.

At the annual meeting of the After-care Association last week, Dr. G. H. Savage said he wished that all asylums were called sanatoria, that instead of the term "idiot" that of "feeble-minded" should be substituted, and that the word "lunatic" were abolished in favour of "mentally disordered."

Great alarm has been caused in Pavia by an outbreak of small-pox in the neighbouring town of Casteggio. The disease is of the most virulent type. All schools and refuges have been closed, and general compulsory vaccination has been ordered.

The memorial stones of a new ward were laid on the 7th inst. at the Accident Hospital, Mansfield. The need of increased hospital accommodation for this district has been seriously felt for some time, owing to the rapid growth of the neighbourhood. The extension will add 25 beds to the hospital, and will include increased kitchen accommodation.

The Central Poor-law Conference was opened at the Guildhall, London, on Tuesday. Mr. Geoffrey Drage, Chairman of the Exmouth Committee of the Metropolitan Asylums Board, in his presidential address referred to the Royal Commission on the Poor-law and said it was hoped that the result of its deliberations would be the codification of the Poor-law, and a definite pronouncement in favour of a greater uniformity of both central and local administration.

The Governor-General of Canada, in addressing the students of the McGill University Medical Society last week, referred to the movement requiring manufacturers of patent medicines to specify on their bottles or packages the ingredients of which their medicines are prepared, and especially their alcoholic strength. He stated that he himself had obtained an analysis of one medicine which was largely advertised in Canada and the United States, and that it was found to contain 40½ per cent. of alcohol.

An address has been presented to the Lord-Lieutenant of Ireland by the fellows and members of the Royal Academy of Medicine, which was founded for the purpose of amalgamating all the Dublin societies of standing that were cognate to the various branches of medicine and surgery. Lord Aberdeen said, in reply, that it was a gratifying token of their success, that the medical societies of London were preparing for a movement similar to that which resulted in the formation of the Academy.

Some three months ago the medical officer of the workhouse at Aberystwyth resigned his post after fifteen years of service, having unsuccessfully applied for an increase of salary. During his tenure of office the late medical officer received the munificent salary of £30 a year, out of which he was required to attend upon and provide medicines for an average of forty inmates. Having failed to find any medical man willing to fill the office under the same conditions, the offended Aberystwyth Guardians have not only failed to make proper provision for the medical care of the sick poor under their charge, but they have deliberately attempted to oust their late medical officer from some other appointments which he holds.

